# Nitridated Ca_2_NaMg_2_V_3_O_12_: Eu^3+^ Vanadate Garnet Phosphor-in-Glass

**DOI:** 10.3390/ma13132996

**Published:** 2020-07-06

**Authors:** Damian Pasinski, Jerzy Sokolnicki

**Affiliations:** Faculty of Chemistry, University of Wroclaw, Joliot-Curie 14 Street, 50-383 Wroclaw, Poland; damian.pasinski@chem.uni.wroc.pl

**Keywords:** vanadate garnet, europium, nitridation, phosphor-in-glass

## Abstract

In this study, Ca_2_NaMg_2_V_3_O_12_ coordination compound undoped and doped with Eu^3+^ obtained in the air and ammonia atmosphere by solid-state reaction was investigated. Ca_2_NaMg_2_V_3_O_12_: Eu^3+^ obtained in the ammonia atmosphere was then investigated as a phosphor-in-glass (PiG). Annealed Ca_2_NaMg_2_V_3_O_12_: Eu^3+^ phosphor forms a single phase with the cubic garnet structure and *Ia3d* space group. Nitridation in ammonia causes a widening of a VO_4_^3−^ group emission band on the low energy side and it red-shifts by 20 nm. The emission of Eu^3+^ is extended as compared to the non-nitridated phosphor. Ca_2_NaMg_2_V_3_O_12_: Eu^3+^ after nitridation shows higher emission quantum yield (QY): 49 vs. 45 and lower correlated color temperature (CCT) and 4179 vs. 4998 as compared to phosphor without nitridation. The QY for PiG is 55. The thermal stability of nitridated phosphors is superior to phosphor obtained in the air atmosphere and is further enhanced for PiG.

## 1. Introduction

One way to obtain white light is to use a phosphor-converted light-emitting diode (pcLED) and blue or ultraviolet pumping LED [1]. The use of UV LEDs as pumping diodes is often a preferred alternative to blue diodes. This applies to cases where the conversion of electrical power into optical power is more efficient for UV diodes than blue ones. In addition, if the pumping diode spectrum is not part of the emission spectrum, any spectral shifts of its band do not affect the pcLED emission spectrum, provided that the phosphor excitation spectrum has a flat characteristic around the pumping diode emission. pcLED photometric parameters, such as correlated color temperature (CCT) and color rendering index (CRI), depend only on the phosphor used or the combination of phosphors. It should be mentioned that obtaining high a CRI in combination with low CCT using a blue LED and a single phosphor seems problematic. For this reason, phosphors are sought that effectively convert UV LED radiation into visible light. 

Vanadates exhibit intensive broadband emissions from 400 nm to over 700 nm under UV excitation [2]. They correspond to the charge transfer (CT) transition from the oxygen 2p orbital to the 3d orbital of V^5+^ in the VO_4_^3−^ group with T_d_ symmetry. This broadband luminescence covers almost the entire visible spectrum, which is very beneficial for obtaining phosphors with high CRI. [3]. Another feature of vanadates is the energy transfer between VO_4_^3−^ and activator ions. Ca_2_NaMg_2_V_3_O_12_ phosphors doped with Eu^3+^ show characteristic broadband emission of VO_4_^3−^ and the narrow line emission of Eu^3+^ in the red region. The luminescent intensity of Eu^3+^ increases due to the energy transfer VO_4_^3−^→Eu^3+^ compared to the emission of the directly excited Eu^3+^ ion. Furthermore, the emission of Eu^3+^ can also supplement the deficiency of the red component in the emission of the VO_4_^3−^ group. At present, there are many reports about similar luminescence systems such as Ca_2_NaMg_2_V_3_O_12_: Eu^3+^ [4], Ca_2_NaMg_2_V_3_O_12_: Sm^3+^, Dy^3+^ [5], and Ca_2_NaMg_2_V_3_O_12_: Sm^3+^ [6]. Setlur et al. observed significant thermal quenching and changes in the emission color of Ca_2_NaMg_2_V_3_O_12_: Eu^3+^ at higher temperatures due to energy migration and transfer to nonradiative traps and Eu^3+^ within the host lattice [7]. These results showed that Ca_2_NaMg_2_V_3_O_12_ is an efficient host lattice. Xie et al. synthesized Eu^3+^-doped Ca_5_Mg_4_(VO_4_)_6_, NaCa_2_Mg_2_V_3_O_12_, KCa_2_Mg_2_V_3_O_12_, and NaSr_2_Mg_2_V_3_O_12_ by the solid-state reaction method [8]. The Eu^3+^-doped phosphors showed higher quantum efficiency than the corresponding undoped ones. Recently, Kim et al. prepared nano-sized Ca_2_NaMg_2−x_V_3_O_12_:xEu^3+^ red phosphors by solution combustion method and then studied the effect of Eu^3+^ content on the photoluminescence properties of the Ca_2_NaMg_2−x_V_3_O_12_:xEu^3+^ phosphors [9]. In turn, Yang et al. analyzed energy transfer mechanisms, energy transfer efficiency, and critical distance (R_c_) of VO_4_^3−^→Eu^3+^ in Ca_2_NaMg_2_V_3_O_12_: Eu^3+^ and showed that the emitting color can turn from blue-green to near white light [10].

It is well known that phosphors used in lighting technology are exposed to thermal attack due to the excitation by light sources with high radiation density and high power, which often leads to the thermal degradation of resins and phosphors as well [11]. One way to solve this problem is to disperse the phosphor in the glass matrix to obtain a phosphor-in-glass (PiG) composite. The glass has higher thermal conductivity and higher endurance against high-power light irradiation than their organic resin counterparts [12,13]. Usually, glass has a low melting point, so that PiG can be fabricated at low temperatures. Another way to improve the thermal stability of the phosphor is to replace some of the oxygen atoms in the oxide matrix with nitrogen atoms so that the oxynitride is formed. Since nitrogen is less electronegative than oxygen, metal–nitrogen bonds have a greater degree of covalency than metal–oxygen bonds. The higher negative charge of nitrogen ions, in turn, causes the active ion to be affected by the stronger crystal field. Both of these effects result in the red-shift of emission bands and improved thermal stability of phosphor [14,15,16]. 

In this work, we examine the influence of both the above-mentioned treatments (nitridation and dispersion in glass) on the spectroscopic and thermal properties of the Ca_2_Na Mg_2_V_3_O_12_: Eu^3+^ phosphor.

## 2. Materials and Methods 

Garnet phosphor powders with formula Ca_2_NaMg_2_V_3_O_12_ and Ca_2_NaMg_2−x_V_3_O_12_:xEu^3+^ were prepared using high-temperature solid-state synthesis. V_2_O_5_ (99.99%, Sigma-Aldrich, Darmstadt, Germany), MgO (99.99%, POCH, Avantor Performance Materials Poland S.A., Gliwice, Poland), CaCO_3_ (99.99%, Alfa Aesar, Ward Hill, MA, USA), Na_2_CO_3_ (99.9%, Chempur, Piekary Slaskie, Poland), and Eu_2_O_3_ (99.999%, Stanford Materials, Lake Forest, CA, USA) were used in stoichiometric amounts. Powders were obtained in the air and ammonia atmospheres at 850 °C for 10 h. Reducing atmosphere was created by the decomposition of melamine to ammonia. Melamine is one of the representatives of triazines where amine groups replace hydrogen atoms from the aromatic ring. The content of nitrogen in melamine is as high as 66.6 mol.%. 

Glasses were synthesized by the conventional melt quenching method at 1400 °C for 1 h. High purity raw material with a composition of 60% SiO_2_ + 25% Na_2_O + 9% Al_2_O_3_ + 6% CaO was used to form a glass frit. After melting, the glass was toughened and then ground to powders with a particle size below ~50 μm. Then, 0.5 g of the glass powders was thoroughly mixed with phosphors and packed into discs with diameters of ~10 mm. The mixing ratio of glass to phosphor was 8:2. The green bodies with glass were fired at 550 °C for 30 min.

The X-ray diffraction (XRD) patterns were measured with a D8 Advance (Bruker) diffractometer (Billerica, MA, USA) using CuKα1 radiation (λ = 1.54056 Å) filtered with Ni. The diffractograms were recorded over the range of 2θ 10–90°. Photoluminescence (PL) and PL excitation (PLE) spectra, as well as decay times, were recorded using an FSL980 spectrofluorometer from Edinburgh Instruments, (Livingston, GB) at 298 K. As an excitation source, a 450 W Xenon lamp was used. The resolution of the measurements was about 0.25 nm. A TEM image was obtained with a FEI Tecnai G2 20 X-TWIN high-resolution transmission electron microscope with LaB6 cathode, FEI Eagle 2K CCD camera, energy dispersive spectroscopy (EDS) detector, and STEM detector, FEI Company, Hillsboro, OR, USA.

## 3. Results and Discussion

**Figure 1:**
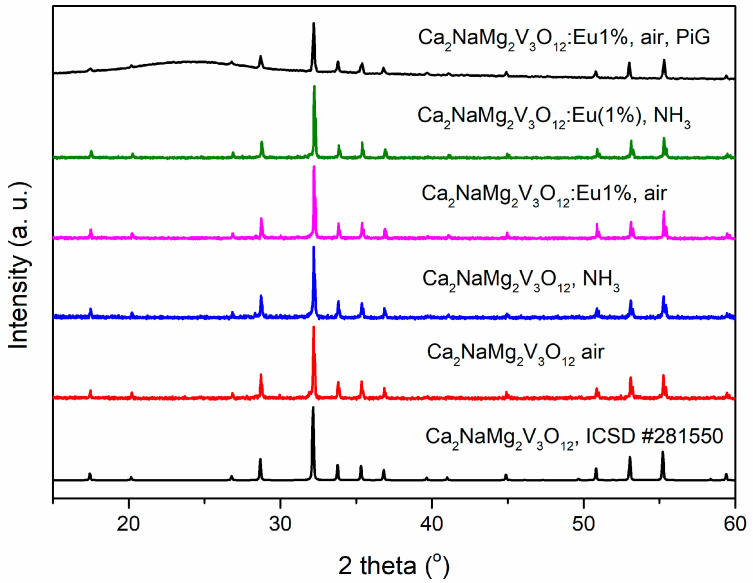
X-ray diffraction (XRD) patterns of undoped Ca_2_NaMg_2_V_3_O_12_ and doped with 1% of Eu^3+^ obtained in different atmospheres and as phosphor-in-glass (PiG) along with the standard card of Ca_2_NaMg_2_V_3_O_12_ (ICSD no. 281550).

Figure 1 presents X-ray powder diffraction patterns of undoped Ca_2_NaMg_2_V_3_O_12_ and doped with 1% of Eu^3+^ obtained in different atmospheres and Ca_2_NaMg_2_V_3_O_12_: Eu (1%) in the glass along with the standard card of Ca_2_NaMg_2_V_3_O_12_ (Inorganic Crystal Structure Database (ICSD) no. 281550). The patterns can be indexed as a cubic garnet structure belonging to the space group of *Ia3d.* No impurity phase was observed in the patterns. It is therefore clear that the Eu^3+^ activator ions have built into the Ca_2_NaMg_2_V_3_O_12_ crystal lattice by substituting Ca^2+^ or Na^+^ ions and that the reaction has been stoichiometric. Only a small shift of diffraction bands towards bigger angles is observed due to the substitution of smaller Eu^3+^ (0.95 A) for bigger Ca^2+^ (0.99 A) or Na^+^ (1.02 A) sites [17]. The diffraction bands of Ca_2_NaMg_2_V_3_O_12_ obtained in the NH_3_ atmosphere shift towards smaller angles. This is due to the lower electronegativity and the larger N^3−^ ion radius compared to O^2−^, which causes M-N ion bonds to be longer than M-O ion bonds. Thus, partial substitution of N^3−^ instead of O^2−^ should cause the average Ca/Na-O binding length to increase. For this reason, a clear shift of the diffraction lines for Ca_2_NaMg_2_V_3_O_12_-NH_3_ towards smaller angles should be expected. This fact may indirectly confirm the incorporation of nitrogen in the phosphor structure. For the Ca_2_NaMg_2_V_3_O_12_ obtained in the NH_3_ atmosphere and doped with Eu^3+^, the shift is not observed due to the compensatory effect. 

In Ca_2_NaMg_2_V_3_O_12_, the alkali and first alkaline earth metal (Ca) ions are found in eight-fold dodecahedral sites with D_2_ symmetry. The second alkaline earth metal Mg^2+^ is in a six-fold octahedral site. The metal ion V^5+^ completely occupies the four-fold T_d_ site in the garnet structure. 

**Figure 2:**
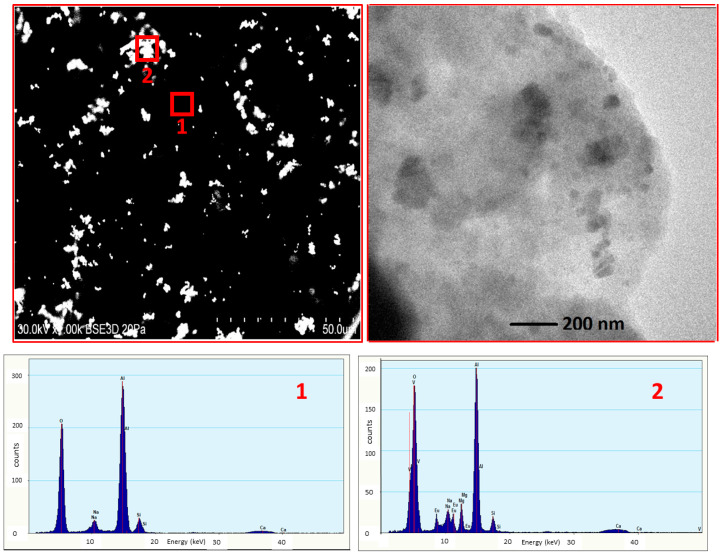
Black/bright field scanning electron microscopy (SEM) images of Ca_2_NaMg_2_V_3_O_12_: Eu^3+^ in glass with energy dispersive spectroscopy (EDS) for the glass region, marked as “1” and for the phosphor region, marked as “2”.

Figure 2 shows a scanning electron microscopy (SEM) image of Ca_2_NaMg_2_V_3_O_12_: Eu^3+^ in glass with the energy dispersive spectroscopy (EDS) for each phase. The Ca_2_NaMg_2_V_3_O_12_: Eu^3+^ particles exist uniformly in the glass matrix with diameters ranging from several hundred nanometers to several micrometers. The EDS clearly shows the Ca_2_NaMg_2_V_3_O_12_: Eu^3+^ and glass matrix phase with their characteristic compositions. The glass phase (1) shows the presence of O, Na, Al, Si, and Ca, while the region enriched in V, Mg, and Eu (2) represents the phosphor. These results show that any interfacial phases between the glass matrix and phosphor are difficult to find, suggesting that the interaction between the two phases may be insignificant and the phosphor particles and the glass matrix can coexist well.

**Figure 3:**
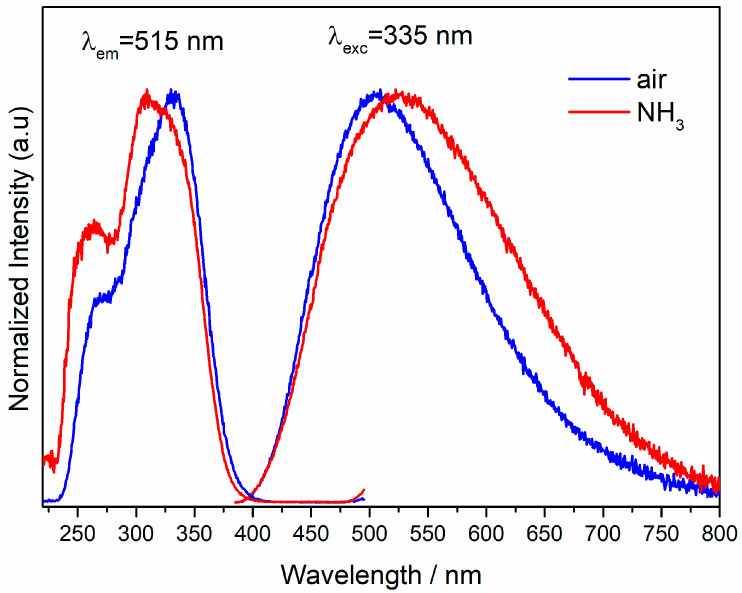
Room temperature excitation and emission spectra of Ca_2_NaMg_2_V_3_O_12_ obtained at different atmospheres.

Figure 3 shows the emission and excitation spectra of the undoped Ca_2_NaMg_2_V_3_O_12_ phosphor obtained in different atmospheres. The pure host lattice obtained in the air atmosphere has a blue-green broadband emission with a peak at 515 nm while the emission of the sample obtained in the NH_3_ atmosphere is red-shifted about 20 nm. In addition, the latter band is widened at the low energy side. 

The luminescence is attributed to the transitions from ^3^*T*_2_ and ^3^*T*_1_ excited states to the ^1^*A*_1_ ground state in VO_4_^3−^ group. The intense and broad excitation spectra consist of the bands centered at 275 and 335 nm, which correspond to the charge transfer from the ^1^A_1_ ground state to the ^1^T_2_ and ^1^T_1_ excited states in VO_4_^3−^ group [9]. The maximum of the excitation band of the nitridated sample is blue-shifted about 15 nm. The red-shift of the emission maximum and the blue shift of the absorption maximum of the nitridated sample can be explained by the fact that in the ammonia atmosphere, nitrogen atoms replace the part of oxygen atoms of the vanadate group. Since the Me-N bond is longer than the M-O bond, the T_d_ symmetry around the V^5+^ ions lowers. This has undoubtedly an impact on the energy levels resulting from the splitting of the 3d multiplet by the crystal field.

Additionally, the lower electronegativity of nitrogen relative to oxygen and the larger negative charge of nitrogen cause stronger crystalline fields around the V^5+^ ions. Consequently, according to the spectroscopic results, the energy of emitting levels decreases, and excited levels increase. The presence of vanadate groups with different O/N ratios would be responsible for broadening the emission range.

Unlike the emission spectra, changes in the excitation spectra depending on the heating atmosphere are negligible.

**Figure 4:**
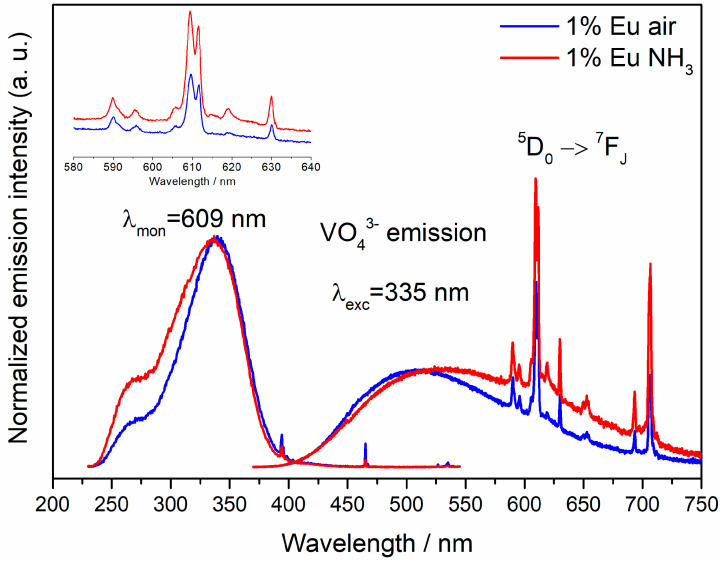
Luminescence spectra excited at 330 nm and luminescence excitation spectra monitoring the emission at 609 nm of Ca_2_NaMg_2_V_3_O_12_: Eu (1%) obtained in the air (red traces) and NH_3_ (blue traces).

Figure 4 presents the luminescence and luminescence excitation spectra of Ca_2_NaMg_2_V_3_O_12_: Eu (1%) obtained in various firing atmospheres. Eu^3+^-doped samples show the narrow emission lines between 580 and 720 nm due to the transitions from the excited ^5^D_0_ state to the stark components of ^7^F levels. These emission lines overlap with a broad emission of the VO_4_^3−^ group. As already shown earlier, with the increase of the Eu^3+^ concentration, the VO_4_^3−^ emission band disappears completely, and the spectrum is dominated by the Eu^3+^ emission [9]. This demonstrates the very efficient VO_4_^3−^→Eu^3+^ energy transfer. We did not repeat this experiment because many authors already confirmed it.

In the aluminum and gallium garnets (e.g., YAG) the emission due to ^5^D_0_→^7^F_1_ transition dominates the spectra because the Eu^3+^ ion occupies a site that has an approximate center of symmetry [18]. In the Ca_2_NaMg_2_V_3_O_12_ garnet, the emission due to the ^5^D_0_→^7^F_2_ transition gives a higher intensity than the other Eu^3+^ emissions in these V-garnets. The enhancement of the ^5^D_0_→^7^F_2_ transitions is a result of the change in the local D_2_ site symmetry, which arises from the replacement of divalent Ca^2+^ by monovalent Na^+^. Charge compensation effects can also play a role. A similar explanation can be made assuming that Eu^3+^ is on the octahedral Mg^2+^ site, as suggested by Kim et al. [9]. Probably, however, Eu^3+^ is on both Ca^2+^/Na^+^ and Mg^2+^ sites at the same time [7]. The introduction of nitrogen into the structure further disturbs the symmetry around the Eu^3+^ ion, which causes that the Eu^3+^ emission from the sample obtained in the ammonia atmosphere is more intense than from the sample obtained in the air atmosphere.

The thermal quenching of the luminescence is important for applications. Setlur et al. [7] observed significant thermal quenching and changes in the emission color of Ca_2_NaMg_2_V_3_O_12_: Eu^3+^ at higher temperatures. This was attributed to VO_4_^3−^ energy migration at high temperatures, leading to a higher probability for energy transfer to lattice defects or Eu^3+^ ions. 

**Figure 5:**
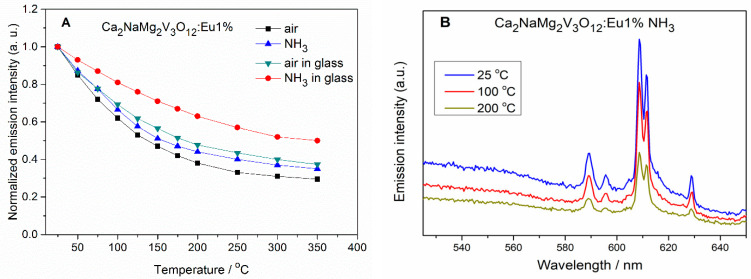
(**A**) Temperature dependence of the integrated emission intensity of Ca_2_NaMg_2_V_3_O_12_: Eu (1%) obtained in different atmospheres and as respective PiG phosphor. (**B**) Temperature dependence of the Eu^3+^ emission intensity for three different temperatures.

The intensity of the emission from Ca_2_NaMg_2_V_3_O_12_: Eu^3+^ was measured as a function of temperature. In Figure 5 the results are shown. It could be observed that the thermal stabilities of Ca_2_NaMg_2_V_3_O_12_: Eu^3+^ obtained in the NH_3_ atmosphere and the same phosphor incorporated into the glass are superior to that of Ca_2_NaMg_2_V_3_O_12_: Eu^3+^ obtained in the air atmosphere. The temperature of 50% quenching of emission intensity for the PiG is around 350 °C, so it is higher than for the sample obtained in the air (around 135 °C). This thermal phenomenon could be ascribed to the improvement of the stability of vanadate structure by incorporation of nitrogen atoms and besides, a glass carrier has stiffened the structure. It should also be emphasized that we did not observe the change in emission color as the temperature changed. The emission intensity of the VO_4_^3−^ and Eu^3+^ groups decreased to the same extent (see Figure 5B). This is understandable because energy transfer from the VO_4_^3−^ group excites the Eu^3+^ emission. Along with the temperature quenching of this emission, the transfer of energy to Eu^3+^ also decreases. 

The thermal quenching energy barrier for phosphors investigated can be estimated using the relationship between the quenching temperature *T*_0.5_ and the energy barrier *∆E* proposed by Dorenbos [19]:

*∆E* = *T*_0.5_/680 eV (1)

where *T*_0.5_ is the temperature at which the emission intensity has dropped to 50% of the initial intensity. *∆E* for Ca_2_NaMg_2_V_3_O_12_: Eu obtained in air (*T*_0.5_ = 135 °C) and nitridated Ca_2_NaMg_2_V_3_O_12_:Eu PiG (*T*_0.5_ = 350 °C) are 0.198 eV and 0.515 eV, respectively. This shows the scale of improving the phosphor thermal stability after nitridation and preparation as PiG.

**Figure 6:**
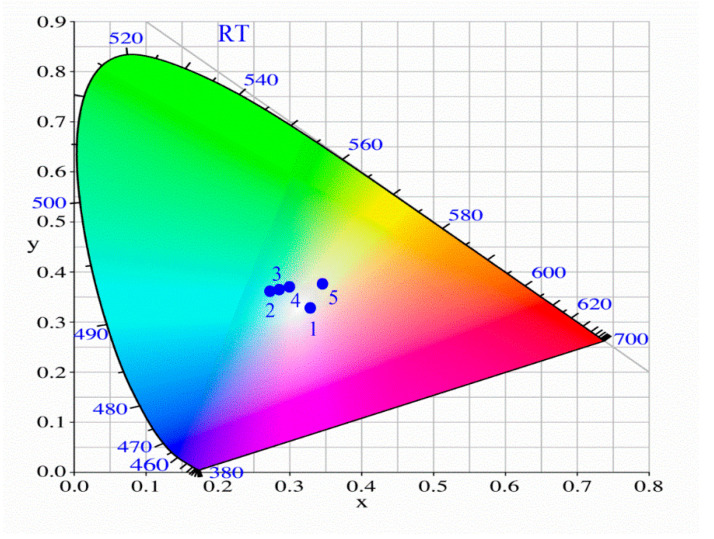
Color coordinates of Ca_2_NaMg_2_V_3_O_12_ phosphors obtained in the air (2) and NH_3_ (3) atmospheres; color coordinates of Ca_2_NaMg_2_V_3_O_12_: Eu (1%) phosphors obtained in the air (4) and NH_3_ (5) atmospheres. 1: white point.

The x and y values of the International Commission for Illumination (CIE) chromaticity coordinates, the color rendering index (CRI), and the correlated color temperature (CCT) values for all phosphors were determined from corresponding emission spectra. The decay times for VO_4_^3−^ and Eu^3+^ emissions were also determined. All the results are presented in Table 1 and their visualization in Figure 6.

For vanadate obtained in the air atmosphere, the CRI is 71. After nitridation, it increases to 78, due to red-shifting and widening of the VO_4_^3−^ emission. Doping with Eu^3+^ ions increases the contribution of red color in the total emission so that the CRI increases to 81 (phosphor without nitridation) and 90 (nitridated phosphor). This last value allows the use of phosphor for internal lighting. With the increase of CRI, the CCT decreases to 4179, the value corresponding to the warmer white color. At the same time, quantum yield (QY) grows in the same order from 36 for vanadate obtained in the air atmosphere to 49 for vanadate obtained in the ammonia atmosphere and doped with Eu^3+^. After the incorporation of this phosphor into the glass, its QY increases to 55, which is the result of the stiffening of the phosphor structure by the glass. The other photometric parameters for PiG are the same as for the phosphor introduced into the glass.

The decay time of vanadate emission is 5.9 µs, the same as recorded by Dhobale and colleagues [5] while of Eu^3+^ emission is approximately 1 ms, which confirms that Eu^3+^ is on non-centrosymmetric sites.

The energy transfer efficiency (*η*_T_) for the non-radiative energy transfer that occurs between (VO_4_)^3−^ and Eu^3+^ can be estimated using the following equation [20]:

*η*_T_ = 1 − *τ*_x_/*τ*_0_ (2)

where *τ*_x_ and *τ*_0_ are the lifetimes of VO_4_^3−^ in the presence and absence of Eu^3+^, respectively. The VO_4_^3−^ to Eu^3+^ energy transfer efficiency calculated using Equation (1) equals 8.5% when the Eu^3+^ content is 1 mol.% and 41.0% when its content is 8 mol.% (concentration for the most effective transfer, *τ* = 3.5 µs). The respective values for nitridated phosphors are: 9.1% and 45.0%.

This value may be however overestimated. Please note that QYs for Eu^3+^-doped phosphors are larger than for non-doped ones. Similar results were obtained by Xie et al., who observed an increase in QYs after doping vanadate with Eu^3+^. They also showed that this trend continues after subtracting Eu^3+^ emissions that overlap with VO_4_^3−^ emissions. This result denies the occurrence of energy transfer from VO_4_^3−^ to Eu^3+^, which is actually being observed. A possible explanation for this phenomenon is the assumption that Eu^3+^, located near the VO_4_^3−^ group, lowers the symmetry of V^5+^ surroundings. This makes the transition probability between the excited and ground states in doped vanadates higher than in non-doped ones. Consequently, the QYs for the former are also higher. At the same time, luminescence lifetimes could be shortened, affecting the values of energy transfer efficiency.

## 4. Conclusions

Ca_2_NaMg_2_V_3_O_12_ undoped and doped with Eu^3+^ was obtained by solid-state reaction in air and ammonia atmospheres. It was shown that nitridation causes the incorporation of nitrogen in the phosphor structure, which changes its physical and chemical properties. The lower electronegativity of nitrogen in relation to oxygen and its higher effective charge cause the emission band of the VO_4_^3−^ group to red-shift and widen, and the absorption band is blue-shifted. The symmetry of the Eu^3+^ ion environment also decreases, which leads to an increase in Eu^3+^ emission. At the same time, the emission QY of the phosphor and its thermal stability increases, while CCT is reduced. The Ca_2_NaMg_2_V_3_O_12_: Eu^3+^ phosphor prepared as PiG shows an even higher QY and thermal stability of the emission intensity. It should be noted that these treatments (nitridation and PiG) allowed to significantly improve the optical parameters of the phosphor under investigation. 

## Figures and Tables

**Table 1:** Color coordinates (x, y), color rendering index (CRI), correlated color temperature (CCT), quantum yield (QY), and decay times *τ* (µs) for Ca_2_NaMg_2_V_3_O_12_ and Ca_2_NaMg_2_V_3_O_12_: Eu samples obtained at different conditions. λ_exc_ = 335 nm.

Phosphor	x Coord.	y Coord.	CRI	CCT	QY	*τ* (µs)
Ca_2_NaMg_2_V_3_O_12_-air	0.27	0.37	71	6908	36	5.9
Ca_2_NaMg_2_V_3_O_12_-NH_3_	0.28	0.38	78	5434	40	5.6
Ca_2_NaMg_2_V_3_O_12_:Eu-air	0.30	0.38	81	4998	45	5.4 998.0 (Eu^3+^)
Ca_2_NaMg_2_V_3_O_12_:Eu-NH_3_	0.35	0.38	90	4179	49	5.1 980.0 (Eu^3+)^
Ca_2_NaMg_2_V_3_O_12_:Eu-NH_3_ glass	0.35	0.38	90	4179	55	5.9 990 (Eu^3+^)
